# Screen-based sedentary behaviour and adiposity among school children: Results from International Study of Childhood Obesity, Lifestyle and the Environment (ISCOLE) - Kenya

**DOI:** 10.1371/journal.pone.0199790

**Published:** 2018-06-28

**Authors:** Lucy-Joy M. Wachira, Stella K. Muthuri, Sophie A. Ochola, Vincent O. Onywera, Mark S. Tremblay

**Affiliations:** 1 Department of Physical and Health Education, Kenyatta University, Nairobi, Kenya; 2 African Population and Health Research Center, Nairobi, Kenya; 3 Department of Foods, Nutrition and Dietetics, Kenyatta University, Nairobi, Kenya; 4 Department of Recreation Management and Exercise Science, Kenyatta University, Nairobi, Kenya; 5 Children’s Hospital of Eastern Ontario Research Institute, Ottawa, Ontario, Canada; Montclair State University, UNITED STATES

## Abstract

**Background:**

High levels of sedentary behaviours have been independently associated with several negative health indicators, including obesity. Screen time (ST) is often used as a contributing measure of sedentary time. It is recommended that children spend no more than 2 hours on recreational sedentary screen-based activities daily. We describe screen-based sedentary time of urban school children and examine the associations between body mass index (BMI) and percent Body Fat (%BF) with ST levels.

**Methods:**

Data were collected from 563 children aged 9 to 11 years attending 29 non-boarding primary schools in Nairobi, Kenya, as part of the International Study of Childhood Obesity, Lifestyle and the Environment (ISCOLE). Data were analysed to test for associations between ST and sex, type of school attended (public verses private), Socioeconomic status, adiposity, and access to electronic devices. We also assessed participants’ ST on school and weekend days.

**Results:**

Of the participants recruited, 15.5% had high ST levels, 67.9% spent no more than 2 hours in recreational screen activities on school days while 74.2% did not meet the guidelines on weekend days. Participants sex was associated with daily ST (t = 3.5, p<0.001), ST on the weekend (t = 3.9, p <0.001) and total ST per week (t = 3.5, p<0.001) with males having higher ST than females. ST was associated with type of school for daily ST (t = 3.6, p <0.001), ST on the weekend (t = 4.5, p<0.001) and total ST per week (t = 3.6, p<0.001) where private schools pupils had higher ST. ST was not associated with BMI. ST was not associated with %BF except on weekend days (p = 0.038) where those classified as overfat/obese (fat) had higher ST.

**Conclusions:**

A large proportion of children spend more time than recommended on screen activities particularly on weekend days. Strategies to improve healthy living should focus on the reduction of sedentary ST for school-aged children.

## Introduction

Public health agencies globally are giving more attention to the increasing evidence that supports sedentary behaviour as a distinct health concern, in addition to getting populations to meet physical activity (PA) recommendations [1]. Despite the perception that children are ‘naturally' active [2], they have been found not to accumulate recommended levels of PA for health, and also spend a significant amount of time in sedentary activities. A sedentary lifestyle is widely recognized as a contributor to the development of obesity [3, 4, 5], and is also associated with decreased physical fitness, self-esteem and academic performance, and increased aggression in children [1]. Sedentary lifestyle often begins early in life, and can persist throughout the life course, ultimately leading to a loss of health and productivity.

Evidence suggests that the amount of time one spends in sedentary behaviour is associated with elevated health risk, independent of level of PA [6]. Sedentary behaviour is categorized to include sedentary screen-based behaviours (e.g., television (TV) viewing, video games, and computer use) and sedentary non-screen-based behaviours (e.g., sitting at school or in a car) [7]. One important factor in exploring sedentary lifestyles in children is determining the time they spend engaging in screen-based activities such as watching television, playing video games and operating a computer [8], collectively referred to as screen time (ST) [3, 9]. With current trends in use of technology, sedentary time has increased dramatically, with children participating in many activities that require minimal energy expenditure [10]. The growth of electronic media and child focused programming has dramatically increased TV viewership by children, and the advent of video, computer, tablet and internet games, or the growing saturation of cell phones with built-in games, as documented in developing countries, is quickly replacing time that children would have otherwise spent in more physically active pursuits [8]. Although ST serves as a valuable index for a sedentary lifestyle, it accounts for only about a third of total sedentary time (SED) [11], with the rest of the SED being spent in several other activities such as reading, passive transport, or eating [12]. Results from the International Study of Childhood Obesity, Lifestyle and the Environment (ISCOLE) indicated that children around the world averaged 8.6 hours of daily SED [12].

In Kenya, there are no existing guidelines for sedentary behaviour, even as opportunities for screen-based activities are increasing [13]. However, organizations such as the American Academy of Pediatrics (AAP) [14] and Canadian Society for Exercise Physiology (CSEP) [15] have sedentary behaviour guidelines which recommend that children aged 5 to 11 years should not engage in more than 2 hours of recreational ST daily as part of a healthy lifestyle. Further, children should reduce motorized transport, long periods of sedentary sitting and time spent indoors throughout the day in order to gain health benefits [14, 15].

There is evidence that children with the most sedentary ST per day have the highest prevalence of overweight and obesity regardless of age, race/ethnicity, or family income [16, 17]. The omnipresent food advertisements targeted to children expose them to highly-processed convenient foods with high caloric content, excessive fat and sugar, and with little or no micronutrient content [18]. The use of screen-based electronic devices is also positively related to overweight in youth [19, 20, 21, 22, 23, 24]. A systematic review by Marshall and colleagues concluded that although television and video/computer games had a positive relationship with body mass index (BMI), the effects were small and unlikely to be of clinical relevance [21], while a different review by Temmel and Rhodes reported that many studies have linked higher levels of sedentarism with greater body weight or adiposity, particularly in homes with availability of media equipment [25]. The somewhat inconsistent findings may be attributable to environmental variability, methodological differences in the measures and cut-points used, and this offers further support for the need to examine the local Kenyan situation.

Previous studies reported that urban children in Kenya spend more time watching TV, playing video games, and on computers, compared to their rural counterparts [26, 27]. A national report card on physical activity on body weight of Kenyan children and youth showed that urban Kenyan children were more sedentary and were exposed to obeseogenic environments (those that promote the consumption of energy dense foods and discourage PA participation) than their rural counterparts [16]. There is need, therefore, to establish the trends in ST and its relationship with overweight and obesity among children living in urban Kenya.

The main objective of this study was to describe screen-based sedentary activity of urban school children in Kenya, in light of the international sedentary recommendations for this age group, while also examining associations between ST levels and sex, type of school attended (public versus private), socioeconomic status (SES), body weight, and fatness status of study participants.

## Materials and methods

### The ISCOLE study

Data collection was conducted in schools within the City of Nairobi in Kenya, as part of the larger ISCOLE. The ISCOLE was a large multi-national study whose aim was to determine the relationships between lifestyle characteristics and obesity among children with representation from twelve countries (Australia, Brazil, Canada, China, Colombia, Finland, India, Kenya, Portugal, South Africa, United Kingdom, and United States). The study targeted a population of urban school children aged 9 to 11 years. The behavioural characteristics examined included self-reported physical activity, sedentary behaviour, sleep and diet, as well as directly measured adiposity and anthropometry and directly measured movements using accelerometry. More details on the ISCOLE study protocol are provided elsewhere [28].

The ISCOLE-Kenya study protocol was reviewed and approved by Kenyatta University Ethics Review Committee (KU/R/COMM/51/15 –PKU005/104). Permission to conduct the study was granted by the National Council for Science and Technology and by the Nairobi County Council.

### Sampling of schools and participants

The primary sampling framework targeted non-boarding primary schools in Nairobi County. The study engaged a sample of 29 non-boarding primary schools. Schools were first stratified into the two main types of schools; private school (owned and run by private entities) or public school (government owned schools) representing various economic classes of schools. Tuition costs and related expenses informed further classification into low (LSES), middle (MSES) and high (HSES) groups to capture a range of economic classes. The recruitment of schools was also planned by the geographical location of schools to avoid clustering in one jurisdiction of Nairobi City.

The secondary sampling framework consisted of 9 to 11 year-olds from each sampled school. Since there are children of various ages within a single classroom in the Kenyan schools, one classroom with most children closest to age 10 years (i.e., 9 to 11 years) was identified and selected. This was to ensure minimal disruption of learning of children from multiple classes since data collection took place during school hours. All children in the selected classroom were invited to participate. Informed consent forms were given to the children to take home for their parent/guardian to complete. Only those whose parent/guardian consented were recruited for the study after they had also assented. The ISCOLE study protocol [28] targeted a minimum sample of 500 children per study site. Out of the 632 children recruited in Nairobi, Kenya, 563 children had all primary data required collected (age, sex, weight, and height), comprising the final sample.

### Adiposity (BMI and body fat (%BF))

Height was measured with shoes removed using a Seca 213 portable stadiometer (Seca, Hamburg, Germany), with the participant’s head in the Frankfurt plane, and after a deep inspiration. The measurement was repeated, and the average of the two used for analyses (a third measurement was obtained if the first two measurements were greater than 0.5 cm apart, and the average of the two closest measurements was used in analyses). The participants’ weight and body fat were measured using a portable Tanita SC-240 Total Body Composition Analyzer (Tanita Corporation of America, Arlington Heights, Illinois). The participant’s outer clothing, heavy pocket items, shoes and socks were removed before taking measurements. Two measurements were obtained, and the average was used in analyses (if the first two measurements were more than 0.5 kg or 2.0% apart, for weight and percentage body fat, respectively, a third measurement was obtained and the closest two were averaged for analyses).

The directly measured weight and height measurements were used to calculate BMI (kg/m^2^), with the WHO reference of BMI-for-age and sex [29] used to categorize participants based on the following percentiles: 5th percentile and below (underweight); from the 5th percentile up to and including the 85th percentile (normal weight); from the 85th percentile up to and including the 95th percentile (overweight); and above the 95th percentile (obese). The Tanita SC-240 Body Composition Analyzer references for percent body fat (%BF) were used to categorize participants into underfat (below the healthy body fat range); healthy fat (within the healthy body fat percentage range for one’s age/sex); overfat (above the healthy range) and obese [30]. The Tanita SC-240 Body Composition Analyzer has acceptable accuracy for estimating %BF when compared with dual-energy X-ray absorptiometry, thus supporting its use in field studies [31].

### Screen-based sedentary behaviour

Information on sedentary screen-based behaviours was obtained using a self-administered questionnaire (ISCOLE Diet and Lifestyle questionnaire) from the participating child. Use of self-reported methods for quantifying ST has acceptable reliability and validity in children [32]. Several of the questions related to sedentary behaviour were obtained from the U.S. Youth Risk Behaviour Surveillance System [33]. Data on electronics in the child’s bedroom was collected through the ISCOLE Neighborhood and Home Environment Questionnaire (filled by the parent/guardian). Table 1 presents questions on the types of screen based electronic devices that participants had access to. Four questions sought information on the amount of time spent in sedentary screen based activities. Participants were asked to indicate how many hours they watched television in a typical school day and weekend day. They were also asked to indicate the number of hours they spent playing video or computer games or using a computer for something that was not school work on a typical school day and weekend day. Information on eating while watching television was also collected to explore snacking behaviours during sedentary screen-based activities. The Neighborhood and Home Environment Questionnaire was completed by the parent/guardian at home soon after the recruitment of participants while the Diet and Lifestyle questionnaire was completed by the participating child during the ISCOLE school visit on the same day when anthropometric measurements were taken. Data collection was conducted throughout the year in 2012.

**Table 1:** Self-report questions and the list of possible answers.

**1. Please tell me what you do**							
	**I do not**	**< 1 hour**	**1 hour**	**2 hours**	**3 hours**	**4 hours**	**5 or more hours**
On a school day, how many hours do you watch TV?	O	O	O	O	O	O	O
On a school day, how many hours do you play video or computer games or use a computer for something that is not school work?	O	O	O	O	O	O	O
On a weekend day, how many hours do you watch TV?	O	O	O	O	O	O	O
On a weekend day, how many hours do you play video or computer games or use a computer for something that is not school work?	O	O	O	O	O	O	O
**2. How many times** **a week** **do you usually eat the following food items while watching television?**							
	**Never**	**Less than once a week**	**Once a week**	**2–4 days a week**	**5–6 days a week**	**Once a day, every day**	**Every day, more than once**
Potato crisps, peanuts or groundnuts	O	O	O	O	O	O	O
Fried food such as chicken wings, chicken fingers, cheese, etc.	O	O	O	O	O	O	O
Cookies, biscuits, chocolate or candy bars	O	O	O	O	O	O	O
Ice cream	O	O	O	O	O	O	O
Fast foods such as chips, pizza, hamburgers, etc.	O	O	O	O	O	O	O
**3. Please indicate whether the following are** **in your child's bedroom**.						**Yes**	**No**
TV						O	O
Computer						O	O
Video game system (Play-station, Xbox, etc.)						O	O
**4. Does your child have the following items for his/her own use?**						**Yes**	**No**
Cell phone or walk-man radio						O	O
Hand-held videogame players (Game Boy, Sony Play station etc.)						O	O

### Screen time

Screen-time variables used in this study were derived from the following information: 1) the number of hours spent on screen-based activities per day on school days, and 2) the number of hours spent on screen-based activities per day on weekend days. The resulting continuous variable data were: average ST on a school day, average ST on a weekend day, average daily ST and the total number of hours in a week on ST behaviours. In addition, participants were categorised into those who met ST guidelines of ≤2 hours/day [34] and those who did not meet the guidelines based on their total average daily ST.

In order to classify the participant’s ST into levels of engagement (low, low-moderate, moderate-high and high), a score was assigned for each response from questions on ST levels for watching TV on school days and on weekend days and ST levels for playing video/computer games/use of computer not for school work on school days and on weekend days. A total score was then computed. Equal quartiles for the scoring were created and used to categorize the participants’ scores into ST levels as follows: “low,” (1^st^ quartile) “low-moderate,” (2^nd^ quartile) “moderate-high,” (3^rd^ quartile) and “high” (4^th^ quartile).

### Data analysis

Statistical Package for Social Sciences (SPSS) computer software (version 17, SPSS Inc. Chicago, Illinois) was used for data analysis. Chi-square tests were used to determine the relationship between individuals’ adiposity and sex and type of school attended, ST categories and sex and type of school attended, ST levels and SES and adiposity, and ST levels and access to electronic devices. These analyses were conducted independently in their specific clusters. Where multiple comparisons were done, confidence intervals were considered and adjustments of P-values using Bonferroni method to address statistical implications of multiple analyses. Odds ratios were generated from the binary (binomial) logistic regression to explain the relationship between the study variables. Independent t-test analysis and one-way ANOVA was used to compare means of continuous variables including average ST on a school day, average ST on a weekend day, average daily ST and the total number of hours in a week on screen-based behaviours by sex, type of school attended and adiposity, independently. A p-value <0.05 was considered significant.

## Results

### Adiposity (BMI and %BF)

Table 2 summarizes the characteristics of sampled schools and the study participants as well as associations between variables from descriptive analysis. The majority (73%) of the participants were of normal weight based on BMI-categorization, and 20.8% were classified as overweight/obese. Based on %BF categorization, 44.9% of the participants were in the healthy category while 13.9% were overfat/obese. More females were overweight (BMI) (56.6%) and overfat (%BF) (67.6%) than males. Public schools had a majority of the normal weight (BMI) participants, but also exhibited greater prevalence of underweight (BMI) and underfat (%BF).

**Table 2:** Descriptive characteristics of study participants and schools.

Study Participants N (563*)*		n	%						
**Age**	9 years 10 years 11 years	207 278 78	36.8 49.4 13.9						
**Sex**	Males Females	262 301	46.5 53.5						
**Participants’ SES**	LSES MSES HSES No response	264 177 102 20	46.9 31.4 18.1 3.6						
**Type of School**	Public Private	295 268	52.4 47.6						
**SES of schools**	LSES school MSES school HSES school	158 358 47	28.1 63.6 8.3	**Males** **(n, %)**	**Females** **(n, %)**	**P** **value**	**Private school** **(n, %)**	**Public school** **(n, %)**	**P** **value**
**BMI for age** **ˠ**	Underweight Normal (ideal) Weight Overweight/Obese	38 411 53 61	6.7 73.0 9.4 10.8	19(50) 189(46) 23(43.4) 31(50.8)	19(50) 222(54) 30(56.6) 30(49.2)	**0.827**	4(10.5) 176(42.8) 39(73.6) 49(80.3)	34(89.5) 235(57.2) 14(26.4) 12(19.7)	<0.001*
**Body fatness** ^†^ **(%BF) (n = 543)**	Underfat Healthy (fat) Overfat Obese (fat)	214 253 34 42	38.0 44.9 6.0 7.5	91(42.5) 128(50.6) 11(32.4) 24(57.1)	123(57.5) 125(49.4) 23(67.6) 18(42.9)	**0.090**	67(31.3) 134(53) 27(79.4) 38(90.5)	147(68.7) 119(47) 7(20.6) 4(9.5)	<0.001*
**Overall Screen Time**	Low ST Low-Moderate ST Moderate-High+ High ST	283 193 87	50.3 34.3 15.4	116(44.3) 92(35.1) 54(20.6)	167(55.5) 101(33.6) 33(11.0)	0.002*	118(44.0) 101(37.7) 49(18.3)	165(55.9) 92(31.2) 38(12.9)	0.015*
**ST on School days#**	2 hours or less of Screen Time More than 2 hours of Screen Time	382 181	67.9 32.1	169(44.2) 93(51.4)	213(55.8) 88(48.6)	**0.113**	180(47.1) 88(48.6)	102(52.9) 93(51.4)	**0.740**
**ST on Weekend days#**	2 hours or less of Screen Time More than 2 hours of Screen Time	145 418	25.8 74.2	55(37.9) 207(49.5)	90(62.1) 211(50.5)	0.016*	42(29) 226(54.1)	103(71) 92(45.9)	<0.001*

Cross-tabulation engaging Chi-square analysis.

* represents *p* values significant at 0.05

^†^Denotes proportions may exclude missing data

**Abbreviations**: n = total; ST = screen Time; SES Socio Economic Status (defined by family annual income); LSES = Low Socio Economic Status; MSES = Middle Socio Economic Status; HSES = High Socio Economic Status. School SES defined by schooling costs/incurred expenses.

**ˠ** denotes use WHO reference of BMI-for-age and sex.

#denotes use of Recommendation: American Academy of Pediatrics (2001) and Canadian Society for Exercise Physiology (2011) 5 to 11 year old children should not engage in more than 2 hours of screen time daily.

### Overall time spent in screen-based sedentary activities

The recommendation that children should spend no more than 2 hours in ST daily [14, 15] was used as a benchmark in this study. The results revealed that on average, participants spent about 1.75 hours daily engaging in recreational ST on school days and about 4.25 hours on weekend days. A majority (67.9%) of the participants spent no more than 2 hours in ST on school days while, most (74.2%) of the participants exceeded the guidelines on weekend days. Overall, children were accumulating a lot more recreational ST on weekend days than on school days.

Based on the scoring and classification into either low or high ST levels, results indicate that the majority (84.5%) of the participants were of low ST while 15.5% had high ST. Further analysis based on the three sub-categories of ST (low, low-moderate and moderate-high and high) revealed that about half of the participants were in the low-level of ST category, 34.3% were in the low-moderate level, and 15.4% were in the moderate to high and High level.

### Screen time levels and access to electronic devices

Table 3 presents results of chi-square analysis in determining associations between access to/presence of selected electronic devices in a child’s bedroom and the participant’s ST level. There was a significant association between having electronic devices in a child’s bedroom and ST, except for computers. Among those that had moderate to-high levels of ST, 75.9% had a TV in their bedroom, 13.8% had a computer, 25.3% had a hand-held video game device, 33.3% had a cell phone, and 19.5% had a non-hand held video game system (e.g., Play-station, Xbox). Assessing the use of various electronics in a week, 76.7% of those who engage in TV viewing over the weekend recorded higher levels of ST.

**Table 3:** Association of ST level and access/use of electronic devices.

	Response	Screen time categories			
		Low	Low-moderate	Moderate - high + High	
***Use of the Electronic devices***					
**TV viewing**	School days	310(55.1%)	76(13.5%)	177(31.4%)	
	Weekend days	86(15.3%)	45(8.0%)	431(76.7%)	
**Computer use, playing video/computer games/use**	School days	421(74.8%)	68(12.1%)	74(13.1%)	
	Weekend days	292(51.9%)	60(10.7%)	211(37.4%)	
***Access to Electronic devices***					**X** **P value**
**TV in participant bedroom**	Yes	245(86.6%)	148(76.7%)	66(75.9%)	9.643 0.008*
	No	38(13.4%)	45(23.3%)	21(24.1%)	
**Computer**	Yes	25(8.8%)	28(14.5%)	12(13.8%)	7.429 0.115
	No	253(89.4%)	164(85.0%)	72(82.8%)	
**Hand-held video game system**	Yes	37(13.1%)	39(20.2%)	22(25.3%)	11.899 0.018*
	No	240(84.8%)	153(79.3%)	62(71.3%)	
**Cell phone**	Yes	47(16.6%)	45(23.3%)	29(33.3%)	15.267 0.004*
	No	229(80.9%)	147(76.2%)	55(63.2%)	
**Video game system (non-hand held e.g. Play station)**	Yes	20(7.1%)	24(12.4%)	17(19.5%)	14.981 0.005*
	No	257(90.8%)	168(87.0%)	67(77.0%)	

Cross-tabulations of variables with Chi-square analysis, all data are frequency and (%). Categorization of ST score: “low,” (1^st^ quartile) “low-moderate,” (2^nd^ quartile) “moderate-high,” (3^rd^ quartile) and “high” (4^th^ quartile).

* represents *p* values significant at 0.05

### Eating while watching television

Analysis of associations between consumption of selected foods while watching television and BMI status indicated that many participants reported eating potato crisps, peanuts, fried foods, cookies and biscuits, ice-cream and fast food while watching television although there was no significant association between these eating behaviours and being overweight/obese.

### Association between ST and sex, type of school and SES

Chi-square analysis was used to determine associations between overall ST and sex, type of school attended and SES (Table 4). Results indicate a significant association between the overall ST levels and sex (χ ^2^ = 12.036, p = 0.002) and school type (χ ^2^ = 8.340, p = 0.015) but no association with SES. Male participants were 2.1 times more likely to have higher ST levels than female participants (OR = 2.1; 95% CI: 1.32–3.37; p = 0.002). Private school participants were found to be 1.5 times more likely to have high ST than public school participants.

**Table 4:** Association of overall ST levels and participant’s sex, school type, SES, BMI and %BF status.

Variables		Screen time categories				Chi-square (P value)
		Low 283(50.3%)	Low-moderate 193(34.3%)	Moderate high +High 87(15.4%)	Total^†^ 563(100%)	
**Child’s Sex**	**Male**	116(44.3%)	92(35.1%)	54(20.6%)	262(46.5%)	12.036 (0.002)*
	**Female**	167(55.5%)	101(33.6%)	33(11.0%)	301(53.5%)	
**School Type**	**Private**	118(44.0%)	101(37.7%)	49(18.3%)	268(47.6%)	8.340 (0.015)*
	**Public**	165(55.9%)	92(31.2%)	38(12.9%)	295(52.4%)	
**SES Categories**	**LSES**	145(54.9%)	80(30.3%)	39(14.8%)	264(46.9%)	8.821 (0.184)
	**MSES**	82(46.3%)	69(39.0%)	26(14.7%)	177(31.4%)	
	**HSES**	50(49.0%)	36(35.3%)	16(15.7%)	102(18.1%)	
**BMI status**	**Underweight**	24(8.5%)	9(4.7%)	5(5.7%)	38(6.7%)	7.488 (0.112)
	**Normal**	205(72.4%)	149(77.2%)	57(65.5%)	411(73.0%)	
	**Overweight/Obese**	54(19.1%)	35(18.1%)	25(28.7%)	53(9.4%)	
**Body fatness status**^†^ **(%BF)**	**Underfat**	113(39.9%)	64(33.2%)	37(42.5%)	214(38.0%)	6.486 (0.166)
	**Healthy (fat)**	129(45.6%)	94(48.7%)	30(34.5%)	253(44.9%)	
	**Overfat / Obese (fat)**	35(12.4%)	25(13.0%)	16(18.4%)	34(6.0%)	

Cross-tabulations of variables, all data are frequency and (%). Categorization of ST score: “low,” (1^st^ quartile) “low-moderate,” (2^nd^ quartile) “moderate-high,” (3^rd^ quartile) and “high” (4^th^ quartile).

^†^Denotes that proportions may exclude missing data. Compared column proportions and adjusted P-values using Bonferroni method where the subscript letter denotes columns whose proportions do not differ significantly from each other.

* represents *p* values significant at 0.05

Abbreviations: LSES = Low Socio Economic Status; MSES = Middle Socio Economic Status; HSES = High Socioeconomic Status.

ST was significantly associated with sex when considering daily ST (t = 3.5, p<0.001), ST on the weekend (t = 3.9, p <0.001) and total ST per week (t = 3.5, p<0.001). Males had significantly higher ST daily, on weekends and in total than the females. ST was also significantly associated with type of school for the daily ST (t = 3.6, p <0.001), ST on the weekend (t = 4.5, p<0.001) and total ST per week (t = 3.6, p<0.001). Students from private schools had significantly higher ST daily, on weekends and in total compared to their counterparts in public schools.

### ST variables and BMI and %BF

Chi-square analysis was used to determine associations between overall ST and adiposity (BMI and %BF) as presented in Table 4. Results indicate no significant association between BMI and ST levels. Associations between body fatness and ST levels were also non-significant.

We also examined mean differences between BMI and the means of ST on school days, on weekend days, daily ST and total number of hours in a week of recreational ST. Results presented in Table 5 reveal no significant difference between BMI and the means of ST regardless of sex or school type. There was no significant differences between %BF and total ST per week, ST on school days and daily ST. There was however significant difference for ST on weekend days (F = 3.295, p = 0.038) where those classified as overfat/obese (fat) had significantly higher ST on weekends days compared to those were not.

**Table 5:** Mean differences of ST Variables and BMI and %BF.

	Total no. of hours/week in screen activities mean (SD)	Average screen time on a school day mean (SD)	Average screen time on a weekend day mean (SD)	Average daily screen time mean (SD)
**Body Mass Index (BMI)**				
Underweight	5.1 (3.9) ^a^	1.6 (2.1) ^a^	3.5 (2.3) ^a^	1.3 (1.0) ^a^
Normal weight	5.9 (3.7) ^ab^	1.7 (1.8) ^a^	4.2 (2.6) ^ab^	1.5 (0.9) ^ab^
Overweight/ Obese	6.5 (4.1) ^b^	1.9 (2.1) ^a^	4.6 (2.7) ^b^	1.6 (1.0) ^b^
***F***	2.257	0.534	2.828	2.255
***p* values**	**0.106**	**0.586**	**0.060**	**0.106**
**Percent Body fat (%BF)**				
Under fat	5.7 (3.7)^a^	1.7 (1.8)^a^	4.1 (2.6)^a^	1.4 (0.9)^a^
Healthy fat	5.9 (3.7)^ab^	1.7 (1.9)^a^	4.1 (2.5)^a^	1.5 (0.9) ^ab^
Overfat/ Obese (fat)	6.7 (4.0)^b^	1.8 (2.1)^a^	4.9 (2.7)^b^	1.7 (1.0)^b^
***F***	1.993	0.167	3.295	1.990
***p* values**	**0.137**	**0.846**	0.038*	**0.138**

ANOVA with Duncan post hoc tests

^ab^ letters represent homogeneous subsets and means with the same superscript letter in the same column are not significantly different and those with different letters are significantly different.

* represents the *p* values that are significant at 0.05

## Discussion

The main focus of this study was to describe recreational ST of urban school children in Kenya, in light of the international ST recommendations for this age group, while also examining associations between ST levels and sex, type of school attended (public versus private), SES, body weight, and fatness status of study participants. Recreational time spent watching TV or videos, playing video games and using computers, was reported to represent a major source of sedentarism in childhood [25].

The study found that school children in Nairobi reported a lot of ST activity, particularly on weekend days. A majority of the participants spent no more than 2 hours in recreational ST on school days, which is expected due to heavier school work load during the school week. However, about three quarters of participants spent more than 2 hours in recreational ST on weekend days. For children with ST in excess of 2 hours per day, experts [14, 15] recommend that they should start to progressively reduce their ST towards meeting the guidelines. Previous studies conducted in Kenya [13, 27] concluded that children residing in urban areas participated in more ST and were less active than children from rural areas, with one study reporting that 13.1% of urban Kenyan children spent more than 11 hours per week playing screen games [13].

The majority of the participants in this study had low ST levels. These results may appear better compared to higher prevalences reported in other populations, especially in developed countries. However, with the emerging PA transition in Kenya resulting from urbanization and increased access to sedentary leisure-time pursuits, there is a need to attend to the situation before it deteriorates further. About 15.5% had high ST levels and are therefore at danger of consequences of excessive ST. Too much ST is associated with poor dietary habits and an increase in ill health [35], poor fitness, increased childhood obesity, increased caloric intake possibly caused by the outcome of advertising, and reduced resting metabolism [36]. In addition, further analysis in our study revealed that many of the participants ate various energy dense foods and snacks while watching TV. Results indicated that those who had high ST were 2 times more likely to have high consumption of cakes/pastries and fast foods, and 1.8 times more likely to have high consumption of potato crisps. Other studies also reported that children who watched more television consumed higher fat foods and more fast food and soft drinks while watching TV compared to healthier options like fruits and vegetables [37]. The relationship between TV viewing and poor diets in this context should be studied further in this population.

Easy access and presence of screen-based electronic devices such as a TV in the child’s bedroom have been associated with high screen time [14]. Most of the participants who engage in TV viewing, especially over the weekend, had high levels of ST and more than three quarters of those that had moderate-high levels of ST, had a TV in their bedrooms. Previous work has shown that having a TV in the bedroom is associated with higher ST [25], greater risk for obesity [38], higher cardiometabolic risk [39], lower PA [40], and shorter self-reported sleep [41]. Interestingly, we found that recreational ST was present in both children in the LSES and HSES in this population indicating an easy access to and presence of electronics in the child’s life. The large number of participants who reported to having a TV in their bedroom (especially those of LSES) could be for reasons that many children from low SES groups spend the night in the family living room where the TV is located and may therefore have extended time of TV viewing. Findings by Carson and colleagues also noted that it is possible that children of LSES living in low income neighborhoods may engage in more ST compared to children living in higher income neighborhoods because they have fewer resources for after-school programs and recreational facilities in their neighborhoods [9]. In addition, perceived lack of safety in low income neighbourhoods may limit children's outdoor play and increase ST through sedentary indoor activity [9]. Children are also not entirely in charge of their ST usage and the easy access and the more time parents/guardians spend watching television and using other media, the more likely the children are to engage in similar activities [42].

There was a sex difference with ST as well as school type difference with ST, but no difference by SES. Males had significantly higher ST over the weekend, daily average, and in total compared to females. Perhaps the female children in this population have more household responsibilities compared to their male counterparts that takes up much of their time. Previous work consistently reported that boys engage in more ST, and are less likely to meet ST guidelines than girls [25, 43, 44, 45], while girls accumulate more SED than boys [25, 46]. This seems to be the situation in most countries worldwide. Private school participants had significantly higher means in daily and weekend ST than those in public schools. Private school participants were found to be 1.5 times more likely to have high ST than public school participants. Private schools (especially the middle and high cost schools sampled in this study) target more affluent families. Their environments may expose the children to more electronic devices and encourage the engagement in screen-based activities for educational, information technology and entertainment purposes.

Studies have concluded that children with the greatest sedentary ST per day have the highest prevalence of overweight and obesity regardless of age, race/ethnicity, or family income [16, 17]. Some studies have reported that the use of screen-based electronic devices are positively related to overweight in youth [19, 20, 21, 22, 23, 24] and concluded that limiting ST among youth to no more than two hours per day may help prevent overweight/obesity among youth. However, the systematic review by Marshall et al. [21] concluded that although TV and video/computer game use have a positive relationship with BMI, the effects are small and are unlikely to be of clinical relevance. Our study found no association between ST and BMI. The reasons for this may be due to influence of other compensatory pathways including increased energy expenditure through physical activity observed in this sample group where participants also reported to play and walk more and recorded high physical activity levels[47, 48]. Our study also found no significant differences between ST variables and % body fat except on weekend days where those classified as overfat/obese (fat) had significantly higher ST on weekends days compared to those who were not. As the study also found that about three quarters of all participants spent more than recommended ST on weekend days, it is evident that those who are overfat/obese (fat) were more sedentary over the weekend and had significantly higher ST on weekend days compared to the rest. High sedentariness, which is also characterized by increased ST, is known to trigger weight and fatness levels as it contributes to energy imbalance and weight gain which influence the adiposity status of a person [4]. The results of this study, like many other studies [25] contribute to the growing evidence that recreational ST is a factor in childhood obesity.

### Strengths and limitations

ISCOLE examined only child-reported ST. A better approach would be to ask both parents and the children to report on ST. The study was also limited to recreational ST and does not examine other sedentary behaviours such as reading or art work, or ST for school. The major strengths of ISCOLE are associated with the overall study design and administration [28], comprehensive training for all involved in the study, and the use of direct measurement for anthropometric variables.

## Conclusions

Children are spending a lot of time on recreational ST, with many exceeding the recommended guidelines, particularly on weekend days. Generally, most urban children have low ST levels while 15.5% had high ST levels. ST had significant associations with sex, school type attended, but not with SES. The study found no associations between ST and BMI. There was also no significance between ST and %BF except on weekend days which was in the expected direction. Strategies for reducing high ST over the weekend should focus on replacing ST with other activities of healthy living. Strategies should particularly focus on reducing the high ST among males to prevent the potential future health consequences. The study findings can also inform the development of public health messages. Future work may aim at understanding related health effects (both negative and positive) of other, non-screen based sedentary behaviours. Further research should also focus on longitudinal assessments throughout childhood and pubertal stage to establish trends and changes in ST and non-screen-based sedentary behaviours as well as relationships between TV viewing and poor diets.

## Supporting information

S1 File Additional data. (XLSX)
